# Twelve Weeks of Combined Resistance and Aerobic Exercise Improves Cardiometabolic Biomarkers and Enhances Red Blood Cell Hemorheological Function in Obese Older Men: A Randomized Controlled Trial

**DOI:** 10.3390/ijerph16245020

**Published:** 2019-12-10

**Authors:** Sung-Woo Kim, Won-Sang Jung, Wonil Park, Hun-Young Park

**Affiliations:** 1 Physical Activity and Performance Institute (PAPI), Konkuk University, 120 Neungdong-ro, Gwangjin-gu, Seoul 05029, Korea; kswrha@konkuk.ac.kr (S.-W.K.); jws1197@konkuk.ac.kr (W.-S.J.); 2 Department of Physical Education, Korea University, 145 Anam-ro, Seongbuk-gu, Seoul 02841, Korea; wonilpark01@korea.ac.kr; 3 Department of Sports Medicine and Science, Graduate School, Konkuk University, 120 Neungdong-ro, Gwangjin-gu, Seoul 05029, Korea

**Keywords:** combined exercise, cardiometabolic biomarkers, RBC hemorheological parameters, aerobic performance, obese older men

## Abstract

The present study examined the effect of a 12-week combined resistance and aerobic exercise training program on cardiometabolic biomarkers and red blood cell (RBC) hemorheological function in 20 obese older men (mean age: 68.8 ± 0.9 years). Subjects were randomly divided into two groups (exercise intervention [EXP; *n* = 10] and control [CON; *n* = 10]). The EXP subjects performed resistance and aerobic exercise training program three times per week for 12 weeks, and the CON subjects maintained their regular lifestyle during the intervention period. Body composition was estimated using bioelectrical impedance analysis equipment. Cardiometabolic biomarkers (glucose, insulin, homeostasis model assessment-estimated insulin resistance (HOMA-IR), HOMA β-cell function, and leptin) and RBC hemorheological parameters (RBC deformability and aggregation) were analyzed. Percent body fat decreased significantly in the EXP group during the intervention period but increased significantly in the CON group. Insulin increased significantly in the CON group over the 12-week period and both insulin and HOMA-IR were significantly higher in the CON group than in the EXP group at post-test. RBC deformability (RBC EI_3Pa) and aggregation (RBC AI_3Pa) improved significantly only in the EXP group. The present study suggests that combined exercise training can be useful for improving cardiometabolic biomarkers and RBC hemorheological parameters in obese older men and may help prevent metabolic syndrome and cardiovascular diseases.

## 1. Introduction

Red blood cell (RBC) aggregation is a reversible structure that consists of three-dimensional layers of RBCs known as “rouleaux” [1]. The effect of RBC aggregation on vessel resistance, tissue perfusion, and blood flow depends on the vascular area where the RBC aggregation flows [1]. RBC aggregates are typically formed in regions with low shear rates in veins. Thus, increased RBC aggregation can lead to a rapid increase in blood viscosity in these areas. Moreover, RBC deformability is another important factor that influences blood viscosity; it depends on various determinants such as membrane viscoelasticity, internal viscosity, and the surface-area-to-volume ratio [2]. Rigid RBCs are less aggregated than deformable RBCs at low shear rates [1]. At very low shear rates, the loss of RBC deformability results in decreased blood viscosity [3]. A sudden increase in blood viscosity can damage the oxygen supply and microcirculation blood flow to tissues [4], and increased blood viscosity is an important risk factor for cardiovascular diseases (CVD). Increased RBC aggregation and reduced deformability have been reported in diseases such as CVD and type 2 diabetes mellitus (T2DM) [5,6,7]. Moreover, hemorheological modifications in obesity, including confusions in the rheological response of RBCs, have been reported by various studies [8,9,10]. The lipid level and insulin sensitivity are biochemical parameters in blood plasma that can potentially influence the rheological characteristics of the RBCs in obese subjects [11,12,13]. In addition, Connes et al. [14] have reported a positive relationship between RBC deformability and oxygen uptake (VO_2_).

Aging may be linked to a number of health problems, including CVD and T2DM [15,16]. The progression of age-related diseases is caused by factors related to the deterioration of physiological condition, including the accumulation of body fat, metabolic dysregulation of blood lipids, and insulin resistance [17,18,19]. Moreover, physical inactivity negatively affects several health conditions, including obesity, T2DM, metabolic syndrome risk factors, and CVD [20]. However, physical activity is a well-known health promotion strategy to prevent physiological decline with aging.

One meta-analysis reported that exercise significantly improves cardiorespiratory fitness and cardiometabolic biomarkers [21]. Cardiometabolic biomarkers include lipid/lipoprotein markers (e.g., TC, TG, HDL-C, LDL-C, FFA etc.), adipokine/inflammatory markers (e.g., CRP, IL-6, TNF-a, leptin etc.), glucose/insulin metabolism markers (e.g., glucose, insulin, HOMA-IR, HOMA-ꞵ etc.), and haemostatic/thrombotic factors (e.g., endothelin, angiotensin, fibrinogen etc.) [21]. Furthermore, several studies have reported a decrease in fat mass after a period of aerobic exercise with increased insulin sensitivity, resulting in improved glucose tolerance [22]. However, one study reported that regular aerobic exercise, e.g., walking and jogging, did not seem to prevent a substantial loss of muscle mass and strength in older adults [23]. On the other hand, another study reported that resistance training was effective in improving the muscle mass and strength in older adult subjects [24]. Furthermore, it has been reported that resistance exercise does not have a more positive effect on cardiorespiratory and metabolic variables than aerobic exercise [25].

The American College of Sports Medicine (ACSM)’s health and fitness journal reported that “group training,” “high-intensity interval training,” and “fitness programs for older adults” ranked second, third, and fourth as the fitness trends of 2019, respectively [26]. High-intensity interval training is an intense exercise method that results in benefits on cardiorespiratory function and metabolic stimulation in just a short period [27,28]. Previous studies have reported the effects of combined resistance and aerobic exercise training. These studies show a positive impact of combined training on body composition, improvement of aerobic performance, a decrease of cardiovascular risk, and an increase of glucose tolerance and insulin sensitivity [29,30,31,32].

Thus, the combination of resistance and aerobic exercise may be necessary to achieve sufficient health benefits in the older adult. This study aimed to examine the effects of combined resistance and aerobic exercise on physiological parameters related to body composition, cardiometabolic biomarkers, and RBC hemorheological function in obese older men.

## 2. Materials and Methods

### 2.1. Subjects

In this study, 24 sedentary and obese older Korean men (mean age: 68.8 ± 0.9 years) not taking any medication with a body mass index (BMI) ≥ 25 [33] and 30% body fat were selected as subjects. These older Korean men were sedentary subjects with low levels of activity who had not performed any kind of exercise over the last six months. Subjects with the following conditions were excluded from the study: those with any chronic diseases that were not under control (e.g., Alzheimer disease, dementia, arthritis, asthma, diabetes, and epilepsy), those who had undergone retinal laser treatment, those who had a history of acute myocardial infarction, those who had undergone joint replacement or suffered from fracture of the lower limb within the previous six months, and those who showed severe cognitive disturbance. Subjects provided written informed consent and were randomly assigned into a control group (CON) or an exercise intervention group (EXP) using a computerized random number generator. In total, 20 subjects completed the study and only their data were used in the analyses (Table 1). Data from the remaining four subjects were discarded due to withdrawal (*n* = 4). This study was approved by the institutional review board (IRB-201812-HR-288) in Korea and all study procedures were conducted in accordance with the Helsinki Declaration.

### 2.2. Study Design

All 20 subjects participated in a one-day pre-test session. On the testing day, cardiometabolic biomarkers (glucose, insulin, homeostatic model assessment for insulin resistance [HOMA-IR], HOMA for ꞵ-cell function [HOMA-ꞵ], and leptin) and RBC hemorheological parameters (RBC elongation (EI) and aggregation (AI) indices) were measured between 7:00 and 9:00 AM in the rested state after overnight fasting. Thereafter, body composition was measured. Subsequently, the VO_2peak_ was measured to evaluate exercise performance in the afternoon.

After the pre-test, subjects were randomly assigned to one of the two groups according to their initial body composition and VO_2peak_: the 10 subjects in the EXP group performed 12 weeks of combined exercise sessions and a one-day post-test session, and the 10 subjects in the CON group performed 12 weeks of maintaining their normal lifestyle without any intervention and a 1-day post-test session.

The EXP subjects performed the following three kinds of combined exercise interventions for 90–120 min: elastic resistance exercise, aerobic exercise on a treadmill, and aerobic exercise on a bicycle. All exercise interventions were performed at a constant temperature and humidity (22 °C, 60%) for a total of 12 weeks, three times a week, at Kyunghee University in South Korea. All EXP subjects performed elastic resistance training sessions consisting of front squats, incline chest presses, seated rows, push presses, split squats, and pull aparts. All subjects performed three sets of 10–15 repetitions at an exercise intensity ranging from 6–7 on the OMNI-Resistance Exercise Scale of Perceived Exertion (OMNI-RES AM; from 0 = extremely easy to 10 = extremely hard); this range has been reported to correspond to exercise intensity levels ranging from 60% to 70% of 1RM, with a rest for 90 s per set. Elastic resistance training sessions were conducted for approximately 30–40 min. For aerobic exercise on a treadmill and a bicycle, the maximal heart rate (HR_max_) was calculated using the Tanaka formula (208 − [0.7 × age]) in the EXP subjects. They then performed 60 min of aerobic exercise corresponding to 60–70% of HR_max_ (treadmill 30 min and bicycle 30 min). Exercise training was supervised and directed by a licensed instructor.

### 2.3. Anthropometric Characteristics and Body Composition

Body height, body weight, BMI, fat-free mass (FFM), fat mass, and percent body fat were measured using a bioelectrical impedance analysis equipment (Inbody 770, Inbody, Seoul, Korea).

### 2.4. Cardiometabolic Biomarkers

Cardiometabolic biomarkers were analyzed by the Green Cross Medical Foundation (Certified organization in The Korea Society for Laboratory Medicine). Concentrations of the following blood parameters were quantified: glucose, insulin, and leptin. A 6 mL sample of venous blood was collected into a serum separating tube (SST) for serum. Clot formation was ensured in the SST by centrifuging the sample at 3500 rpm for 10 min. Glucose was determined using an enzymatic kinetic assay (Modular PE, Roche, Germany) and insulin was determined using an electrochemiluminescence immunoassay (ECLIA) (Modular E170; Roche, Germany). Leptin was analyzed using a radioimmunoassay method (MERCK, Darmstadt, Germany). HOMA-IR and HOMA-ꞵ were calculated using the following formula; HOMA-IR = [glucose (mg/dL) × insulin (µU/mL)]/405, HOMA-ꞵ = [360 × insulin (µU/mL)]/[glucose (mg/dL) − 63].

### 2.5. RBC Hemorheological Parameters

We measured RBC deformability and aggregation to evaluate RBC hemorheological function. Uyuklu et al. [34] recommended that RBC deformability and aggregation should be analyzed at 25 °C at shear stress of 3 Pa within 4–6 h after collecting blood, so all samples were analyzed within 30 min of their collection at a room temperature of 25 °C using a Rheoscan-D (Rheo Meditech Inc., Seoul, Korea). For RBC EI analysis, the sample was transferred to a 2 mL microfuge tube and then diluted in 700 μL of 5.5% polyvinylpyrrolidone (360 kDa) dissolved in 1 mmol phosphate buffered saline (pH 7.4; osmolality = 300 mOsmol/kg) in a K3EDTAtube (Greiner bio-one, Chon Nuri, Thailand). Then, 0.5 mL of this solution was analyzed using a D-test kit according to manufacturer’s instructions (Rheo Meditech Inc.). For the accuracy of RBC EI measurement, a Lineweaver-Bruke plot model (LB model) was used [35]. For the RBC AI analysis, 8 μL of the blood sample (direct whole blood analysis) was analyzed using an A-test kit according to manufacturer’s instructions (Rheo Meditech Inc., Seoul, Korea).

### 2.6. Aerobic Performance

To assess aerobic performance, VO_2peak_ was measured using the modified BRUCE protocol for graded exercise testing (GXT) on a treadmill using the Vmax-229 breath-by-breath auto metabolism analyzer (SensorMedics, Yorba Linda, CA, USA). Heart rate (HRmax) was measured using a heart rate monitor (Polar RS400, Polar Electro Oy, Kempele, Finland). The individual HRmax was determined as the highest value measured during the GXT. The VO_2peak_ was evaluated as the average of the highest values measured over the last 30 s. subjects were required to reach the following criteria: (1) plateau of VO_2_ achieved, (2) HRmax achieved (220 – age (±10%)), (3) inability to maintain the effort, (4) VCO_2_/O_2_ ratio of > 1:1.

### 2.7. Statistical Analysis

Means and standard deviations (SD) were calculated for each primary dependent variable. Normality of distribution of all outcome variables was verified using the Kolmogorov-Smirnov test. A two-way analysis (‘group’ × ‘time’) of variance with repeated measures on the ‘time’ factor was used to analyze the effects of the training programs on each dependent variable. A partial eta-squared (η*_p_*^2^) was calculated as a measure of effect size. The effect size was computed as partial eta-squared values (η*_p_*^2^; small: ≥0.01, medium: ≥0.06, large: ≥0.14) [36]. Independent and paired t-tests were applied if any significant interaction or main effects were detected. All analyses were performed using Statistical Package for Social Science (SPSS) version 23.0 (IBM Corp., Armonk, NY, USA). The level of significance was set at 0.05.

## 3. Results

### 3.1. Body Composition

Pre- and post-test body composition data for both groups are shown in Table 2. All body composition variables showed a significant interaction (body weight: F = 14.229, *p* < 0.001, η*_p_*^2^ = 0.442; FFM: F = 13.994, *p* < 0.001, η*_p_*^2^ = 0.437; fat mass: F = 87.840, *p* < 0.001, η*_p_*^2^ = 0.830; percent body fat: F = 282.897, *p* < 0.001, η*_p_*^2^ = 0.940). Post-hoc analyses found that the CON group showed a significant decrease in FFM (*p* = 0.013) and increase in fat mass (*p* = 0.001), percent body fat (*p* < 0.001). There was a significant decrease in body weight (*p* = 0.001), fat mass (*p* < 0.001), and percent body fat (*p* < 0.001) in the EXP group.

### 3.2. Cardiometabolic Biomarkers

As shown in Table 3, insulin (F = 9.337, *p* = 0.007, η*_p_*^2^ = 0.342) and HOMA-IR (F = 5.179, *p* = 0.035, η*_p_*^2^ = 0.223) showed a significant interaction. As a result of the post-hoc analyses, insulin increased significantly between the pre- and post-tests in the CON group (*p* = 0.039). However, insulin no significantly between the pre- and post-tests in the EXP group. Insulin was significantly lower in the EXP group at the post-test analysis (*p* = 0.003). Furthermore, HOMA-IR was significantly lower in the EXP group at the post-test analysis (*p* = 0.015).

### 3.3. RBC Hemorheological Parameters

**Figure 1:**
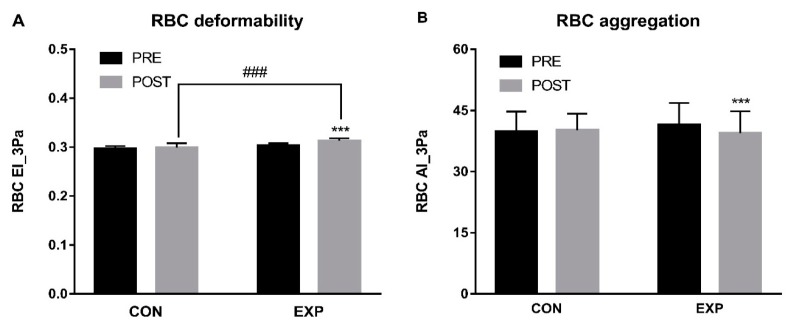
RBC hemorheological parameters before and after the 12-week training program. (**A**) For RBC deformability, statistical analyses reveal an increase between pre- and post-test parameters in the EXP group. (**B**) For RBC aggregation, statistical analyses reveal a decrease between pre- and post-test parameters in the EXP group. RBC = red blood cell, EI = elongation index, AI = aggregation index, CON = control group, EXP = experimental group. Significant difference between pre- and post-tests, *** *p* < 0.001. Significant difference between CON and EXP groups, ^###^ *p* < 0.001.

Figure 1 depicts pre- and post-intervention data for RBC deformability (EI) and aggregation (AI). The repeated two-way ANOVA analyses revealed a significant interaction for RBC EI_3 Pa (F = 4.966, *p* = 0.037, η*_p_*^2^ = 0.216) and RBC AI_3 Pa (F = 18.269, *p* < 0.001, η*_p_*^2^ = 0.504). Post-hoc analyses found that the EXP group showed a significant improvement in RBC deformability (*p* < 0.001) and aggregation (*p* = 0.001).

### 3.4. Aerobic Performance

**Figure 2:**
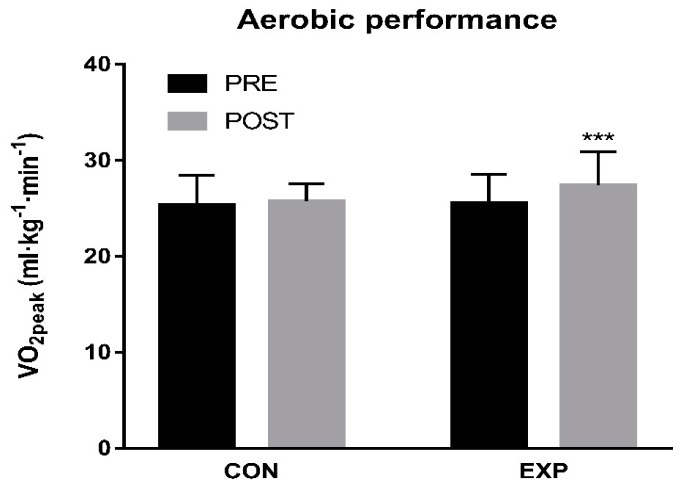
VO_2peak_ before and after the 12-week training program. For aerobic performance, statistical analyses reveal an increase between pre- and post-test in the EXP group. VO_2peak_ = peak oxygen uptake, CON = control group, EXP = experimental group. Significant difference between pre- and post-tests, *** *p* < 0.001.

As shown in Figure 2, there was a significant interaction for VO_2peak_ (F = 5.277, *p* = 0.034, η*_p_*^2^ = 0.227). Post-hoc analyses found that there was a significant improvement in VO_2peak_ in the EXP group (*p* < 0.001).

## 4. Discussion

Recommendations by the ACSM and the American Heart Association to combine aerobic and resistance exercises for overall health extends to obese older adults [37]. The present study examined the effects of a 12-week combined resistance and aerobic exercise program on body composition, cardiometabolic biomarkers, and RBC hemorheological function in sedentary and obese older men. The findings of this study were that combined exercise training reduced body weight, fat mass, percent body fat, leptin levels, and RBC aggregation and increased RBC deformability and aerobic performance. Our results support those of a previous study that reported that exercise training is the most beneficial type of exercise for the frail older adult [38].

Aging is associated with changes in body composition, such as an increase in body fat mass and a decrease in skeletal muscle mass [39]. Previous studies have demonstrated that combined intervention produces the most significant improvements in body composition [29,40,41]. In the present study, the EXP group significantly reduced body weight (−2.16%), fat mass (−7.93%), and percent body fat (−5.91%) over the 12-week intervention, whereas the CON group significantly decreased FFM (−2.16%) and significantly increased fat mass (6.26%), and percent body fat (5.15%). Chen et al. [29] reported that 8 and 12 weeks of combined aerobic and resistance exercise training significantly decreased body weight and percent body fat in older adults. In contrast, the control group showed decreased skeletal muscle mass [29]. Therefore, combined training is considered as a useful exercise strategy to reduce fat mass and percent body fat and maintain skeletal muscle mass in older adults.

Obesity is associated with a chronic inflammatory status and increased risk of cardiometabolic disease [42]. Adipose tissue serves endocrine functions, including the secretion of the proinflammatory cytokine and a role in energy storage [42]. Indeed, the common association between obesity and various comorbid states, including CVD and T2DM, is this state of chronic low-grade inflammation [43,44]. Obesity involves increased adipose tissue, which results in high circulating levels of free fatty acids and inhibits insulin-stimulated glucose uptake [45]. This ultimately leads to increased insulin production and synthesis and elevated plasma glucose levels [45]. The meta-analysis mentioned earlier concluded that exercise training significantly improves the cardiometabolic biomarkers of glucose intolerance and insulin resistance, lipid and lipoprotein metabolism, and systemic inflammation [21]. In the present study, insulin increased significantly in the CON group (9.65%) over the 12-week study period, and the post-test HOMA-IR was significantly higher in the CON group (CON: 1.06 ± 0.12, EXP: 0.93 ± 0.11). However, there was no significant change in glucose levels after the intervention period. Balducci et al. [46] reported that combined exercise training (aerobic: 70–80% of VO_2_max, resistance: 80% of 1RM) for 12 weeks significantly decreased insulin in older adults. Another study showed that after 12 weeks of combined training, insulin and HOMA-IR were significantly lower in an obese female [47]. Another previous study showed that a control group of older females had significantly higher insulin and HOMA-IR following the 12-week study period [48]; however, there was no change in glucose levels in that study, consistent with the present findings. Our study showed that insulin levels decreased without changes in glucose levels, which suggests that glucose was removed from the blood.

Adipose tissue secretes a variety of adipocytokines, including adiponectin and leptin, and also plays a vital role in energy metabolism [49]. The present study showed a greatly decreased plasma leptin level (−24.31%) in the EXP group. Previous studies have shown that various exercises reduce serum leptin levels [50,51]. One meta-analysis reported that the decrease in leptin levels observed after strict exercise training may be due to enhanced leptin sensitivity and may indicate that the body is establishing a new physiological ‘set point’ [50]. In parallel with the suppression of leptin levels, exercise training also improves carbohydrate metabolism parameters [51].

The primary function of RBCs is related to the surrounding micro-circulation tissue, in that they facilitate the exchange of oxygen and carbon dioxide [52]. RBCs need to be modified to circulate through small capillaries that are smaller in diameter than the RBCs themselves [52]. Few studies have been conducted to determine RBC deformability and aggregation improvements in older adults after regular exercise. In the present study, a change in RBC hemorheological parameters (RBC EI_3Pa: 3.31%, RBC AI_3Pa: −4.84%) was observed after 12 weeks of combined exercise training. Regular exercise (aerobic or resistance) usually reduces blood viscosity [53,54]. Exercise training also induces the rheological adaptation of RBCs [54,55]. One study on healthy volunteers who participated in regular exercise training over 12 weeks showed a decrease in blood viscosity and an increase in RBC deformability [56]. In cardiovascular disorders, hemorheological benefits that result from regular exercise training are assumed to contribute to the enhancement of cardiovascular health caused by the training program [57].

Cardiorespiratory fitness is an important component of health-related fitness, which refers to the ability of the respiratory and cardiovascular systems to supply oxygen to muscles during continuous and intense exercise [21]. Cardiorespiratory fitness has also been used as an indicator of regular exercise. In the present study, the EXP group showed significantly increased VO_2peak_ (7.52%) following the 12-week intervention. One meta-analysis reported that a combined exercise program had a moderately positive effect on VO_2peak_ compared to the control group (3.6 mL·kg^−1^·min^−1^) [58]. In the context of combined training, the inclusion of strength training may provide additional benefits, because previous studies have reported improved cardiorespiratory fitness compatibility after strength training that is potentially mediated by increased mitochondrial enzyme activity and capillary density [59,60]. Furthermore, improvements in lower body muscle strength may also lead to increase the time to “all-out” on a graded exercise test, increasing the observed VO_2peak_ [61]. Previous studies have reported that cardiorespiratory fitness is associated with functional capacity and independent living in older adults [58]. We suggest that combined exercise training increases cardiorespiratory fitness, which improves cardiovascular function in older adults.

## 5. Limitation of the Study

In this study, there are some limitations to consider when interpreting results. Although present studies have been designed systematically with randomly controlled experiments, small sample sizes can be limited to check the effects of an exercise intervention on cardiometabolic biomarkers and RBC hemorheological parameters in older men. The appropriate number of subjects may be needed in future studies to access clinical practice. Secondly, the subject’s dietary intake and physical activity were not investigated.

## 6. Conclusions

The present study revealed that a 12-week combined exercise training program reduced percent body fat, and leptin levels and improved RBC hemorheological parameters and aerobic performance in obese older men. We believe that a combined exercise training program could be useful for improving body composition, cardiometabolic biomarkers, and RBC hemorheological function in obese older men, ultimately leading to better health and cardiovascular function in this population.

## Figures and Tables

**Table 1:** Characteristics of the subjects.

Variables	CON (*n* = 10)	EXP (*n* = 10)	*t*-value
Age (years)	68.5 ± 0.85	69.1 ± 0.88	–1.555
Body height (cm)	165.8 ± 4.82	164.1 ± 3.79	0.846
Body weight (kg)	71.6 ± 5.00	70.7 ± 3.84	0.434
BMI (kg/m^2^)	26.0 ± 0.43	26.2 ± 0.48	0.301
Fat free mass (kg)	45.4 ± 3.17	44.8 ± 2.43	0.434
Percent body fat (%)	32.7 ± 1.78	32.4 ± 1.37	0.434

Values are expressed as mean ± standard deviation. CON = control group, EXP = experimental group, BMI = body mass index.

**Table 2:** Changes in body composition before and after the 12-week training program in obese older men.

Variables	CON		EXP		*F*-value (ηp^2^)		
	Pre	Post	Pre	Post	Time	Group	Interaction
Body weight (kg)	71.6 ± 5.00	72.3 ± 5.05	70.7 ± 3.84	69.2 ± 4.09 ***	1.921 (0.096)	0.970 (0.051)	14.229 (0.442) ^†††^
Fat free mass (kg)	45.4 ± 3.17	44.4 ± 3.10 *	44.8 ± 2.43	45.2 ± 2.67	2.379 (0.117)	0.014 (0.001)	13.994 (0.437) ^†††^
Fat mass (kg)	23.5 ± 2.97	24.9 ± 3.14 ***^,^^###^	22.9 ± 2.23	21.1 ± 2.22 ***	1.063 (0.056)	3.337 (0.156)	87.840 (0.830) ^†††^
Percent body fat (%)	32.7 ± 1.78	34.3 ± 1.90 ***^,###^	32.4 ± 1.37	30.4 ± 1.38 ***	1.151 (0.060)	8.577 (0.323) ^†^	282.897 (0.940) ^†††^

Values are expressed as mean ± standard deviation. CON = control group, EXP = experimental group. Significant interaction or main effect, ^†^*p* < 0.05, ^†††^ *p* < 0.001. Significant difference between pre- and post-tests, * *p* < 0.05, *** *p* < 0.001. Significant difference between CON and EXP groups, ^###^ *p* < 0.001.

**Table 3:** Changes of cardiometabolic biomarkers before and after the 12-week training program in obese older men.

Variables	CON		EXP		*F*-value (ηp^2^)		
	Pre	Post	Pre	Post	Time	Group	Interaction
Glucose (mg/dL)	114.38 ± 12.02	116.53 ± 6.63	120.04 ± 7.76	114.32 ± 7.04	0.488 (0.026)	0.354 (0.019)	2.383 (0.117)
Insulin (µU/mL)	3.39 ± 0.36	3.68 ± 0.19 *^,###^	3.43 ± 0.22	3.27 ± 0.20	0.732 (0.039)	4.885 (0.213) ^†^	9.337 (0.342) ^††^
HOMA-IR	0.97 ± 0.20	1.06 ± 0.12 ^#^	1.02 ± 0.13	0.93 ± 0.11	0.000 (0.000)	0.736 (0.039)	5.179 (0.223) ^†^
HOMA-ꞵ	24.53 ± 3.79	24.93 ± 1.71	21.85 ± 1.69	23.16 ± 2.05	1.306 (0.068)	7.533 (0.295) ^†^	0.370 (0.020)
Leptin (μg/L)	22.08 ± 8.82	19.75 ± 6.53	22.56 ± 8.01	17.03 ± 7.00 **	7.459 (0.293) ^†^	0.132 (0.007)	1.232 (0.064)

Values are expressed as mean ± standard deviation. CON = control group, EXP = experimental group, HOMA-IR = homeostatic model assessment for insulin resistance, HOMA-ꞵ = homeostatic model assessment for ꞵ-cell function. Significant interaction or main effect, ^†^ *p* < 0.05, ^††^ *p* < 0.01. Significant difference between pre- and post-tests, * *p* < 0.05, ** *p* < 0.01. Significant difference between CON and EXP groups, ^#^ *p* < 0.05, ^###^ *p* < 0.001.
